# Predictors for low coverage of uptake of second dose of measles vaccine among children in sub-Saharan Africa, 2023: a systematic review and meta-analysis

**DOI:** 10.1080/20523211.2023.2285507

**Published:** 2023-12-07

**Authors:** Tamirat Melis, Ayenew Mose, Yohannes Fikadu, Kassahun Haile, Aklilu Habte, Gemechu Jofiro

**Affiliations:** a Department of Public Health, College of Medicine and Health Science, Wolkite University, Wolkite, Ethiopia; b The Ritchie Centre, Hudson Institute of Medical Research, Clayton, Australia; c Department of Obstetrics and Gynaecology, Monash University, Clayton, Australia; d Department of Midwifery, College of Medicine and Health Science, Wolkite University, Wolkite, Ethiopia; e Department of medical laboratory, College of Medicine and Health Science, Wolkite University, Wolkite, Ethiopia; f Department of Public Health, College of Medicine and Health Science, Wachamo University, Hosanna, Ethiopia; g Department of Nurse, College of Medicine and Health Science, Arsi University, Asela, Ethiopia

**Keywords:** Second dose measles vaccine, utilisation of measles vaccine, vaccine uptake

## Abstract

**Background:**

Measles became a public health important disease in sub-Saharan Africa. World Health Organization recommended measles-containing vaccine dose 2 (MCV2) through routine service delivery. This study aims to determine coverage of second-dose measles vaccination uptake and its predictors among children aged 24–35 months in sub-Saharan Africa.

**Methods and materials:**

We conducted an extensive search of literature as indicated in the guideline of reporting systematic review and meta-analysis (PRISMA). The databases used were PubMed, Google Scholar, and HINARI literature.

**Results:**

The overall uptake of the second dose of measles vaccine uptake was 41% (95% CI: 28.90–53.47). Caregiver's awareness of the importance of the second dose of measles (2.51, 95% CI 1.77, 3.25), educational status of mothers (1.30, 95% CI 1.16, 1.45), distance from vaccination site (1.22, 95% CI 1.12, 1.32), and attending four and above ANC visit (2.72, 95% CI 2.29, 3.15) were determinants for second dose measles vaccine uptake.

**Conclusion:**

Coverage of the second dose of measles uptake in Sub-Saharan Africa was low (41%) which is lower than the recommendation from WHO. Therefore policymakers and stakeholders should increase mother's awareness. Also, special strategies should be developed for those who are far from the vaccination site.

**Abbreviation and acronyms:**

ANC: Ante Natal Care; JBI: Joanna Briggs Institute; MCV1: Measles containing vaccine dose 1; MCV2: Measles containing vaccine dose 2; WHO: World Health Organization

## Background

One of the major causes of death among under-five children is measles, which is a very contagious respiratory disease caused by the measles virus (Griffin et al., 2012; Tatsuo et al., 2000). World Health Organization recommended Measles containing vaccine dose 1 should be given at 9 months of age, and a second dose of measles vaccine at age 15–18 months through routine services strategies, if ≥80% coverage of MCV1 at the national level for 3 consecutive years (Gagneur et al., 2008; Perry et al., 2014; World Health Organization, 2002, 2009a). However, the approach for the introduction of MCV2 was changed in April 2017 and now advises nations to incorporate MCV2 into their national immunisation programmes, irrespective of the degree of MCV1 coverage (World Health Organization, 2017c). In Ethiopia, the second dose of measles vaccination was introduced into the routine vaccination programme on 11 February 2019 to be administered at 15 months of age (Immunization MoHEPo, 2019). In a study conducted in Ethiopia, only 9.1% of children aged 24–35 months received the second dose measles vaccine (Demewoz et al., 2023).

Globally, 207,500 individuals die because of measles, of which, 147 900 (over 70%) occurred in African countries in 2019 (Patel et al., 2020). Sub-Saharan Africa accounts for the highest morbidity and mortality from measles (Onoja & Ajagbe, 2019). Globally, deaths from measles declined by three-fourths from 2000 to 2014 but measles is still considered a public health emergency disease (World Health Organization, 2012). Vaccinating Two doses of measles vaccine for at least 95% of the population are used to effectively prevent the transmission of measles by ensuring herd immunity (WHO/Europe 2015). Measles is serious and fatal in all age groups; however, children younger than 5 years of age are more at risk for complications like ear infections, diarrhea, pneumonia, and encephalitis (swelling of the brain). It can also lead to long-term complications of a fatal disease called sub-acute sclerosing pan encephalitis (Harris et al., 2014; World Health Organization, 2022).

The efficacy of giving measles vaccine dose two is better in preventing the measles disease compared to only one dose of measles (Garly et al., 1999; Makam et al., 2019; Njie-Jobe et al., 2012). Also, giving a second dose of measles is used to eradicate the virus successfully by advancing the coverage of two routine doses of the measles vaccine (Wolfson et al., 2009). African countries incorporated the second dose of the measles vaccine and performed different activities to increase the second dose of the measles vaccine. They form national-level committees. Endorsed MCV2 introduction guideline, strengthened cold chain management, prepared reporting format, Prepared MCV2 monitoring chart, and gave training for stakeholders. However, there were challenges like poor cold chain management, absence of an MCV2 monitoring chart in most African countries, weak supportive supervision, lateness in the introduction and logistic input of MCV2, and health workers not having a clear understanding of the policies regarding MCV2 (Masresha et al. 2018).

The global world faced difficulty in eliminating measles because measles vaccination coverage in Sub-Saharan Africa is low (Goodson et al., 2011; Strebel et al., 2003). Despite, comprehensive activities done to reduce measles mortality, epidemics continue in certain countries (Akalu, 2015; World Health Organization, 2009b, 2010, 2017a).

Therefore, this systematic review and meta-analysis study aims to determine coverage of second-dose measles vaccination and its predictors among children aged 24–35 months in sub-Saharan Africa. This finding is used to be cumbersome for low uptake of second dose of measles vaccine in Sub-Saharan Africa by identifying causes for non-uptake of MCV2. This study was also used as baseline data for researchers and policymakers of the world.

## Methods and materials

### Study design and setting

A systematic review and meta-analysis were conducted to determine the pooled coverage of second-dose measles vaccination and its predictors among children aged 24–35 months in sub-Saharan Africa. We used the Systematic Review and Meta-Analysis (PRISMA) guideline (PTRoSRaM-A).

### Search strategies and sources of information

Databases like PubMed, Scopus, Google Scholar, African Journals Online, and Web of Sciences were used as search engines. Medical Subject Headings (MeSH) and key terms had been developed using different Boolean operators ‘AND’ and ‘OR’. The following search terms were used:- ‘measles second dose’ OR ‘second dose measles vaccine’ OR ‘second dose measles vaccination’ AND ‘associated factor’ OR: ‘determinants’ and (‘Children’ OR ‘children aged 24–35 months’ AND ‘Sub Saharan Africa’ to search under PubMed/Medline search engine. For the other databases we had search using the following search terms ‘second dose measles vaccination and its predictors among children aged 24–35 months’. The search period for this study was from 3 March 2023 to 15 May 2023.

### Eligibility criteria

Studies conducted with the title of second dose measles vaccine coverage and factors associated with it which were done in Sub-Saharan Africa were included. Both published and unpublished including pre-print studies at any time in the English language only were considered. Regarding the study period, there was no restriction. Articles without full abstracts or texts and articles reported out of the outcome interest were excluded

### Outcome measurements

The primary outcome was taking a second dose of measles vaccination. If a child takes a second dose measles vaccine it is considered as ‘vaccinated for the second dose of measles’ and if not considered as ‘Not vaccinated for the second dose of measles vaccine’. The secondary outcome variables that affect second doses of measles vaccination are considered secondary outcomes.

### Data extraction

All articles obtained by searching from different engines were exported to Endnote version X8 software. Then these articles were exported to a Microsoft Excel spreadsheet. A standardised data extraction tool was used for data extraction which incorporates the year of publication, names of the authors, study design, study setting, study region, sample size, and coverage of the second dose of measles vaccination [Using odds ratio with their 95% confidence interval]. Three reviewers extracted the data independently. Then they compile the extracted data. If there is any disagreement, they solve the disagreements by freely discussing and resolving and they reach a common consensus.

### Quality assessment

Study quality was assessed using a standardised tool adapted from the Newcastle–Ottawa quality assessment tool for Crossectional studies which is adapted from the Newcastle–Ottawa Quality Assessment Scale for cohort studies (UNAIDS JUNPoHA, 2014). Three authors (TM, AM, and YF) assessed the quality of the tool by assessing the representativeness of the sample, on respondent rate, determining adequate sample size, and ascertainment of exposure variable with respective points. We summed up the total value for individual study out of ten. Those articles that fulfilled the required criteria were scored value 1 while those that did not fulfil the criteria were scored 0 and considered as having poor quality respectively. No article was excluded from the review because of poor quality.

### Data processing and analysis

Articles searched using different search engines were imported into Endnote Version 6 software and duplicates were removed. Data were recorded in abstraction forms and entered into Stata 14 software for analysis.

Systematic review and meta-analysis were conducted by using STATA 14 software. We pooled the estimate of the total coverage of uptake of second doses of measles vaccine using a random effect model. The overall pooled uptake of second doses of measles vaccine with 95% confidence among children in Sub-Saharan Africa was reported using a forest plot. Cochran statistics and I2 statistics were used to assess the heterogeneity of the study. The level of statistical heterogeneity between studies was assessed using I2 statistics and values of 25, 50, and 75% were considered to represent low medium, and high, respectively. The random-effects model was used for the data identified as heterogeneous during analysis. We performed meta-regression analyses to find the most likely cause of heterogeneity using STATA version 14 statistical software. A funnel plot was used to detect the presence of publication bias.

Subgroup analysis based on the country was done to further pinpoint the potential cause of heterogeneity across the studies. The presence of significant publication bias was measured using the funnel plot and Egger's regression tests (p-value < 0.05 was considered to be suggestive of statistically significant publication bias).

## Results

### Search results and characteristics of included studies

**Figure 1.**
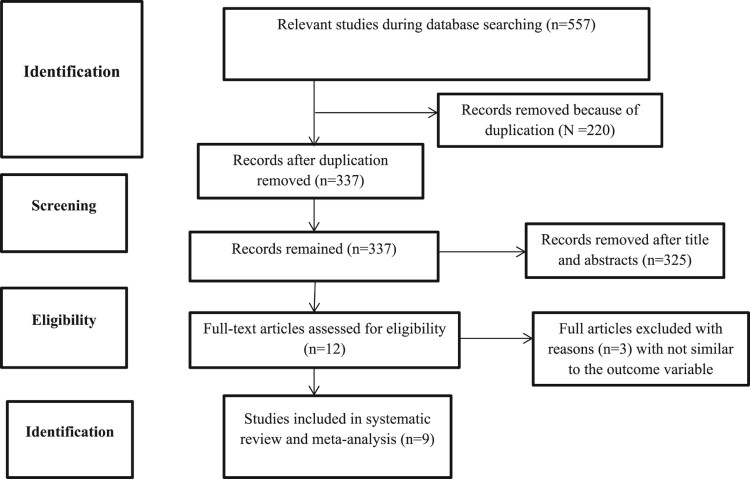
PRISMA flow diagram for the studies screened, reviewed, and included for the study of second dose measles vaccine in sub-Saharan Africa, 2023.

A total of 557 potential studies were found using different search engines, 182 from PubMed engine, 120 from Hinari, 156 from Google Scholar, 96 from African Online Journal and 3 were searched by cross reference. Eight articles that fulfilled the eligibility criteria were included in a systematic and meta-analysis study. We extracted full-text screening after checking the abstract for eligibility (Figure 1)

The total number of study participants from these 9 articles was 20,392. The minimum sample size was 329 (Diress et al., 2020) and the maximum sample size was 15,090 (Zealiyas, 2020). The highest and lowest coverage second doses of measles vaccine uptake were in Burkina Faso and Ethiopia region, which was 62% and 9.8% respectively (Koala et al., 2022; Teshale & Amare, 2023) (See Table 1).

**Table 1.** Characteristics of articles for the study of second dose measles vaccination coverage and its predictor in Sub-Saharan Africa, 2023.

Author, publ. year	Study design	Study region	Study population	Study setting	Sample size	P in (%)	Determinant factors (OR, 95%CI)	quality
Koala et al. (2022)	Crossectional	Burkina Faso	Children 24–35 month	Community-based	329	62	Multiparous mothers (AOR = 2.4, 1.5–4.0).	7
Muluneh et al. (2022)	Crossectional	Ethiopia	Children 24–35 month	Community-based	965	12.36	Not vaccinated for penta 3 (AOR = 0.60;0.37–0.95), educational status (0.51;0.26,0.99)	7
Chilot et al. (2022)	Crossectional	WHO Africa region	Children 24–35 month	Community-based	15,090	44.77	Maternal secondary education [AOR = 1.27, 1.13–1.43)], access health facilities [AOR = 1.21,1.12–1.31)], four and above ANC visit [AOR = 2.75,2.35–3.24)], PNC visit [AOR = 1.13,1.04–1.23)], health facility delivery [AOR = 2.24, 2.04–2.46)]	8
Teshale and Amare (2023)	Crossectional	Ethiopia	Children 24–35 month	Community-based	800	9.84	Mothers 35–49 years (AOR = 0.04, 0.90), being the 4th–5th child (AOR = 4.02;1.45, 11.14) and 6^th^ and above child (AOR = 4.12; 1.42, 13.05)	7
Tadesse et al. (2022)	Crossectional	Ethiopia	Children 24–35 month	Community-based	410	42.5	Average time mothers had been waiting for vaccination at the health facility [AOR = 3.68;1.33, 10.23)]; awareness about vaccine-preventable diseases (AOR = 4.15;1.53, 11.26); and awareness of recommended measles doses (AOR = 17.81;3.91, 81.22)	8
Demewoz et al. (2023)	Crossectional	Ethiopia	Children 24–35 month	Community-based	845	48.1	Mothers’ education status (AOR = 1.91;1.15–3.17), information about MCV2 (AOR = 6.53;4.22–10.08), distance from vaccination site (AOR = 3.56;2.46–5.14), knowledge about immunisation (AOR = 1.935;1.29–2.90), and favourable attitude about immunisation (AOR = 5.19;3.25–8.29)	7
Ogutu et al. (2023)	Crossectional	Kenya	Children 24–35 month	Community-based	417	51.1	Child's birth order (AOR 2.6; 1.33–4.89) and caregiver education (AOR 1.9; 1.10–3.31), number of ANC visits (AOR 2.30; 1.17–4.52); and caregiver not preferring nearby health facility (AOR 2.6; 1.32–5.22)	8
Mamuti et al. (2022)	Crossectional	Kenya	Children 24–35 month	Community-based	536	56.2	Caregiver’s knowledge on the number of MCV scheduled doses (AOR = 5.73;3.48–9.45), birth order was 5th born (AOR = 0.5, 0.22–0.95)	7
Magodi et al. (2019)	Crossectional	Tanzania	Children 24–35 month	Community-based	442	44.2	unaware of the ages MR2 administration (AOR = 3.50; 1.98–6.21), having MR2 vaccination services offered at the local vaccination station fewer than three days per week (AOR = 1.50; 1.42–5.59), not having the vaccine available during vaccination days (AOR = 3.38; 1.08–10.61), the unwillingness of health workers to open multi-dose vaccine vials for a single child (AOR = 3.80;12-6.79), and long waiting times for vaccination services (AOR = 1.80; 1.08–3.00)	7

### The coverage of second-dose measles vaccination in Sub-Saharan Africa

**Figure 2.**
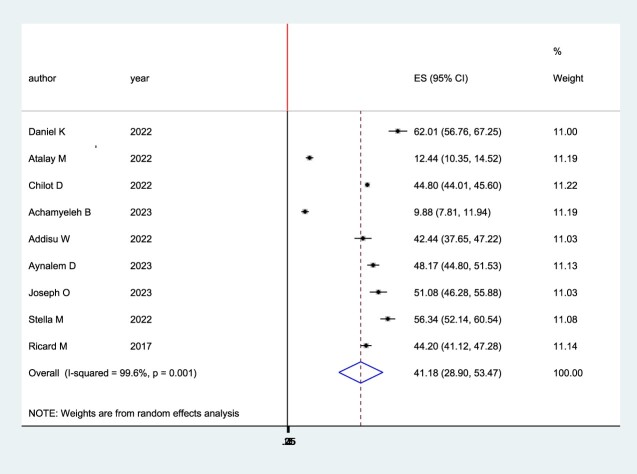
Forest plot for the pooled coverage of second dose measles vaccine uptake among children in Sub-Saharan Africa, 2023.

The pooled prevalence of second-dose measles vaccination in Sub-Saharan Africa was plotted using a forest plot. The pooled prevalence of second-dose measles vaccination in Sub-Saharan Africa was 41% (95% CI: 28.90–53.47) with I2 = 99.6%, p ≤ 0.001 (Figure 2).

### Publication bias

**Figure 3.**
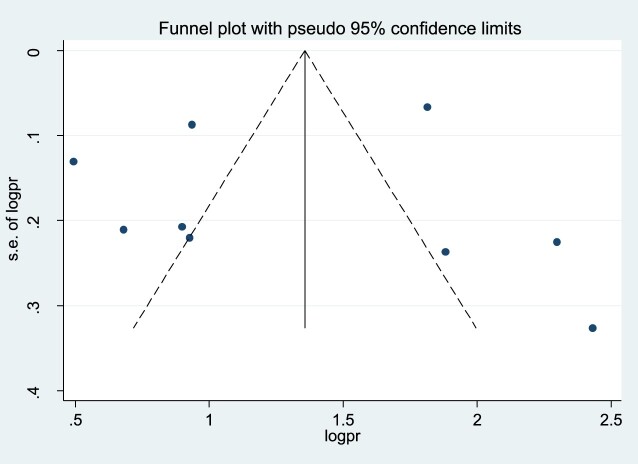
Funnel plot to assess publication bias for the pooled coverage of second dose measles vaccine uptake among children in Sub-Saharan Africa, 2023.

A funnel plot test was used to assess if there is publication bias. As shown in Figure 3, there was no substantial publication bias for the study of second-dose measles vaccination in Sub-Saharan Africa (Figure 3).

### Sensitivity analysis

**Figure 4.**
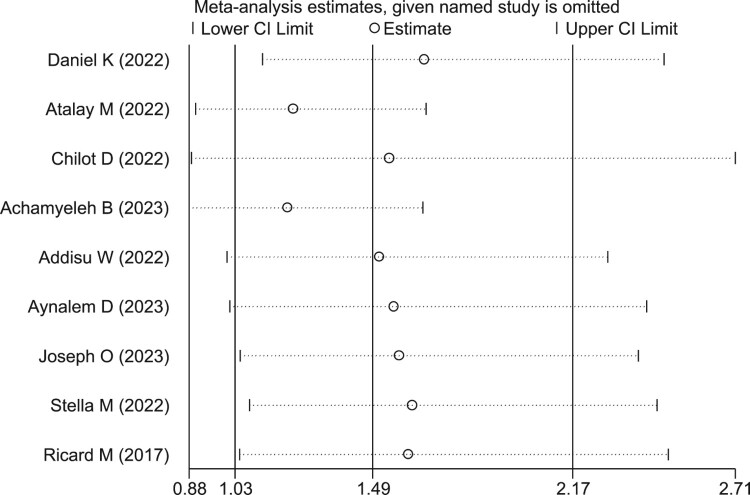
Sensitivity analysis for the study of pooled coverage of second dose measles vaccine uptake among children in Sub-Saharan Africa, 2023.

Sensitivity analysis was done to assess if only a single study influenced the pooled coverage of second-dose measles vaccination in Sub-Saharan Africa. The sensitivity analysis showed the studies were not affected by a single study (Figure 4).

### Subgroup analysis

**Figure 5.**
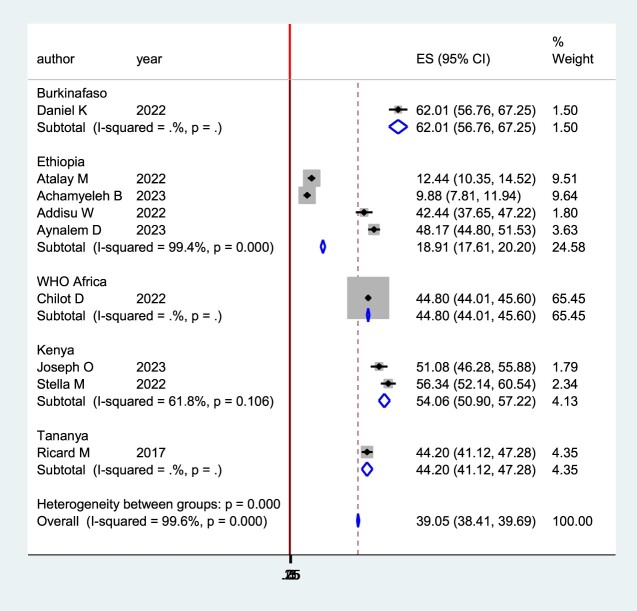
Subgroup analysis by country for the study of coverage of second dose measles vaccine uptake among children in Sub-Saharan Africa, 2023.

The sub-group analysis showed that Ethiopia has the lowest level of second-dose measles vaccine coverage (18%). But, Burkina Faso has the highest level of second-dose measles vaccine coverage (62%) (Figure 5).

### Determinants of second-dose measles vaccine uptake

In this study, Care giver’s awareness of the importance of a second dose of measles for their child, educational status, distance from the vaccination site, and attending four and above ANC visits were determinant factors in systematic analysis for second-dose measles vaccine uptake in Sub-Saharan Africa

Mothers who had a good awareness of the importance of a second dose of measles for their child were 2.5 more likely to vaccinate a second dose of the measles vaccine than their counterparts (AOR: 2.51; 95% CI :(1.77, 3.25). The odds of second-dose vaccine uptake was 2.3 more likely study population that easily accessed to vaccination site when compared to their counterparts (AOR: 1.22; 95% CI :(1.12, 1.32). Mothers who had attended four or more ANC visits were 2.5 more likely to uptake a second dose of the measles vaccine than three or fewer ANC visits (AOR: 2.72; 95% CI :(2.29, 3.15). Mothers who had attended primary or more educational level were 1.3 more likely to vaccinate their second dose of measles vaccine for their child than their counterparts (AOR: 1.3; 95% CI :(1.16, 1.45).

## Discussion

The second-dose measles vaccination uptake and related determinants among children in Sub-Saharan Africa were assessed in this study using a primary study. It showed that in sub-Saharan Africa, the pooled level of second-dose measles vaccine uptake was 41%, which indicates that 59% of children in the region are still not immunised. This coverage is lower than the recommendation of the World Health Organization which states that all children should receive second doses of the measles vaccine (World Health Organization, 2019). Also, it is lower than the study conducted in China which is (93.3%) (World Health Organization, 2017b), and Japan (90.8%) (Sugishita et al., 2019). The justification for this low coverage of MCV2 in sub-Saharan Africa might be due to low awareness of mothers on the importance of a second dose of measles vaccine, poor accessibility of children for a second dose of measles vaccine, mothers’ attitude toward vaccines, non-availability of vaccines in health institutions are obstacles for non-uptake for the second dose of measles vaccines.

Subgroup analysis by study country was done (Figure 5). The sub-group analysis showed that Ethiopia has the lowest level of second-dose measles vaccine coverage (18.91% (95% CI: 17.61–20.20. But, Burkina Faso has the highest level of second-dose measles vaccine coverage (62.01% (95% CI: 56.76–67.25). The justification for significant differences between these countries might be variations in health systems and the level of activities implemented for the awareness creation of caregivers on the importance of a second dose of measles vaccine for their child.

Care giver’s awareness of the importance of a second dose of measles for their child, educational status, distance from vaccination site, and attending four and above ANC visits were determinant factors in systematic analysis for second dose measles vaccine uptake in Sub-Saharan Africa.

Mothers, who had a good awareness of the second dose of the measles vaccine, were more likely to vaccinate their children than their counterparts. This study is supported by a study conducted in northwest Ethiopia (Demewoz et al., 2023), northern Ethiopia (Tadesse et al., 2022), and Zambia (Nchimunya et al., 2023). The justification might be mothers who had awareness of the second dose of measles know the importance of vaccine-preventable disease increase and the effects of non-vaccinating their child. Therefore, they had better intentions to vaccinate their children (Nchimunya et al., 2023).

Mothers who attend primary and above were more likely to vaccinate their children than compared to mothers who attend less than primary education. This study is consistent with studies in northwest Ethiopia (Demewoz et al., 2023), the Ethiopian demographic health survey (Muluneh et al., 2022), the World Health Organization African Region (Chilot et al., 2022), and Kenya (Ogutu et al., 2023). The justification might be that educated mothers had better access to knowledge about the importance of a second dose of measles vaccine for their child. Increased knowledge of mothers on the importance of vaccine-preventable diseases increases mothers’ belief and attitude toward vaccine-preventable diseases which in turn increases the probability of vaccinating their children (Makokha et al., 2015; Nchimunya et al., 2023).

Children who are residing near vaccination sites were more likely to receive a second dose of measles vaccine than their counterparts. This study is supported by different studies (Chilot et al., 2022; Demewoz et al., 2023; Koala et al., 2022; Okwaraji et al., 2012; Russo et al., 2015; Wiysonge et al., 2012). This might be mothers who are far from the vaccination site lack of travel cost, motion sickness, travel phobia, and lack of transport to reach the vaccination site.

Attending four and above ANC visits is the determinant factor for the uptake of a second dose of the measles vaccine. Those mothers who had attended four and above ANC visits were more likely to vaccinate their children than mothers who had attended less than four ANC visits. This study has a similar finding to a study conducted in (Abadura et al., 2015; Chilot et al., 2022; Landoh et al., 2016; Laryea et al., 2014; Ogutu et al., 2023). The possible justification might be fully ANC attended mothers get full information related to maternal and child care. They get information about the importance of vaccine-preventable diseases and the schedules for when to vaccinate their child.

## Conclusion

The pooled coverage of the second dose of measles uptake was 41% which is lower than the recommendation from WHO as all children should take a second dose of the measles vaccine. However, 60% of children in Sub-Saharan Africa did not receive a second dose of the measles vaccine. Care giver's awareness of the importance of a second dose of measles for their child, educational status of mothers, distance from vaccination site, and attending four and above ANC visits were predictors for low coverage of second dose measles vaccine uptake. Therefore, health workers should increase mothers’ awareness by providing counselling services. Special strategies should be developed for those who are far from the vaccination site. Also, policymakers should work on MCV2 should be considered as a routine activity of health workers.

## Supplementary Material

Supplemental Material

## Data Availability

The data is available with no restriction. It could be obtained from corresponding author (Tamirat Melis, Email address: tamiratmelis27@gmail.com3

## Additional file (s1–s3)

**S1.** Prisma checklist for the study of the uptake of the second dose of measles vaccine
**S2.** Newcastle – Ottawa Quality Assessment Scale for Cohort Studies
**S3.** Quality assessment score for individual items for the study of uptake of the second dose of measles vaccine

## References

[CIT0001] Abadura, S. A., Lerebo, W. T., Kulkarni, U., & Mekonnen, Z. A. (2015). Individual and community level determinants of childhood full immunization in Ethiopia: A multilevel analysis. *BMC Public Health*, *15*(1), 1–10. 10.1186/s12889-015-2315-z 26415507 PMC4587824

[CIT0002] Akalu, H. B. (2015). Review on measles situation in Ethiopia; past and present. *Journal of Tropical Diseases & Public Health*, *4*, 2. 10.4172/2329-891X.1000193.

[CIT0003] Chilot, D., Belay, D. G., Shitu, K., Gela, Y. Y., Getnet, M., Mulat, B., Muluneh, A. G., Merid, M. W., Bitew, D. A., & Alem, A. Z. (2022). Measles second dose vaccine utilization and associated factors among children aged 24–35 months in sub-Saharan Africa, a multi-level analysis from recent DHS surveys. *BMC Public Health*, *22*(1), 1–12. 10.1186/s12889-022-14478-x 36371164 PMC9655865

[CIT0004] Demewoz, A., Wubie, M., Mengie, M. G., Kassegn, E. M., Jara, D., Aschale, A., & Endalew, B. (2023). Second dose measles vaccination utilization and associated factors in Jabitehnan District, Northwest Ethiopia. *Dose-Response*, *21*(1), 15593258231164042. 10.1177/15593258231164042 36923301 PMC10009019

[CIT0005] Diress, G., Dagne, S., Alemnew, B., Adane, S., & Addisu, A. (2020). Viral load suppression after enhanced adherence counseling and its predictors among high viral load HIV seropositive people in North Wollo Zone public hospitals, Northeast Ethiopia, 2019: Retrospective cohort study. *AIDS Research and Treatment*, Article ID 8909232, 9. 10.1155/2020/8909232 PMC7191360 32373359

[CIT0006] Gagneur, A., Pinquier, D., Aubert, M., Balu, L., Brissaud, O., De Pontual, L., Le Guen, C. G., Hau-Rainsard, I., Mory, O., Picherot, G., Stephan, J.-L., Cohen, B., Caulin, E., Soubeyrand, B., & Reinert, P. (2008). Kinetics of decline in maternal measles neutralizing serum antibodies in infants in France in 2006. *Clinical and Vaccine Immunology*, *15*(12), 1845–1850. 10.1128/CVI.00229-08 18815232 PMC2593172

[CIT0007] Garly, M.-L., Martins, C. L., Balé, C., da Costa, F., Dias, F., Whittle, H., & Aaby, P. (1999). Early two-dose measles vaccination schedule in Guinea-Bissau: Good protection and coverage in infancy. *International Journal of Epidemiology*, *28*(2), 347–352. 10.1093/ije/28.2.347 10342702

[CIT0008] Goodson, J. L., Masresha, B. G., Wannemuehler, K., Uzicanin, A., & Cochi, S. (2011). Changing epidemiology of measles in Africa. *Journal of Infectious Diseases*, *204*(Suppl_1), S205–SS14. 10.1093/infdis/jir129 21666163

[CIT0009] Griffin, D. E., Lin, W.-H., & Pan, C.-H. (2012). Measles virus, immune control, and persistence. *FEMS Microbiology Reviews*, *36*(3), 649–662. 10.1111/j.1574-6976.2012.00330.x 22316382 PMC3319515

[CIT0010] Harris, J. B., Marta, G. -D., Eggers, R., Brown, D. W., Sodha, S. V., & Centers for Disease Control and Prevention (CDC). (2014). Global routine vaccination coverage, 2013. *Morbidity and Mortality Weekly Report*, *63*(46), 1055. 25412062 PMC5779507

[CIT0011] Immunization MoHEPo. (2019, February 11). Expanded programme on immunization, launched measles vaccine second dose.

[CIT0012] Koala, D., Kleme, M.-L., Ouedraogo, I., Savadogo, I., Ouedraogo, W. T., Ahawo, A. K., Tall, H., & Zoungrana, K. A. (2022). Factors associated with the low immunization coverage in the second year of life in the central region of Burkina Faso. *Fortune Journal of Health Sciences*, *5*(4), 596–602. 10.26502/fjhs.089

[CIT0013] Landoh, D. E., Ouro-Kavalah, F., Yaya, I., Kahn, A.-L., Wasswa, P., Lacle, A., Nassoury, D. I., Gitta, S. N., & Soura, A. B. (2016). Predictors of incomplete immunization coverage among one to five years old children in Togo. *BMC Public Health*, *16*(1), 1–7. 10.1186/s12889-016-3625-5 27618851 PMC5020474

[CIT0014] Laryea, D. O., Abbeyquaye Parbie, E., & Frimpong, E. (2014). Timeliness of childhood vaccine uptake among children attending a tertiary health service facility-based immunization clinic in Ghana. *BMC Public Health*, *14*(1), 1–5. 10.1186/1471-2458-14-90 24476339 PMC3912921

[CIT0015] Magodi, R., Mmbaga, E. J., Massaga, J., Lyimo, D., Mphuru, A., & Abade, A. (2019). Factors associated with non-uptake of measles-rubella vaccine second dose among children under five years in Mtwara district council, Tanzania, 2017. *The Pan African Medical Journal*, *33*. 10.11604/pamj.2019.33.67.17055 PMC6689852 31448029

[CIT0016] Makam, L., Mathew, J., Ratho, R., Dutta, S., Singh, M., Bharti, B., Suri, V., & Massey, D. (2019). *G147 randomized controlled trial comparing anticipated measles vaccination schedules to routine vaccination starting at 9 months in Indian infants*. BMJ Publishing Group Ltd.

[CIT0017] Makokha, F., Wanjala, P., Githuku, J., & Kutima, H. (2015). Uptake of a second dose of measles-containing vaccine among children in Kakamega County, Kenya. *International Journal of Scientific and Research Publications*, *5*(7), 1–4.

[CIT0018] Mamuti, S., Tabu, C., Marete, I., Opili, D., Jalang’o, R., & Abade, A. (2022). Measles containing vaccine coverage and factors associated with its uptake among children aged 24–59 months in Cherangany Sub County, Trans Nzoia County, Kenya. *PLoS One*, *17*(2), e0263780. 10.1371/journal.pone.0263780 35196355 PMC8865666

[CIT0019] Masresha, B. G., Luce, R., Okeibunor, J., Shibeshi, M. E., Kamadjeu, R., & Fall, A. (2018). Introduction of the second dose of measles-containing vaccine in the childhood vaccination programs within the WHO Africa region–lessons learned. *Journal of Immunological Sciences*, S (017), 113–121. PMC6372060 30766972

[CIT0020] Muluneh, A. G., Merid, M. W., Tigabu, B., Ferede, M. G., Kassa, G. M., & Animut, Y. (2022). Less than one-fifth of Ethiopian children were vaccinated for measles second dose; evidence from the Ethiopian mini demographic and health survey 2019. *Vaccine*, *X*(12), 100217. 10.1016/j.jvacx.2022.100217 PMC9486014 36148266

[CIT0021] Nchimunya, M., Chanda, D., & Musenge, E. (2023). Factors contributing to the acceptability of second dose of measles vaccine among children in Livingstone district, Zambia. *Open Journal of Pediatrics*, *13*(2), 220–234. 10.4236/ojped.2023.132028

[CIT0022] Njie-Jobe, J., Nyamweya, S., Miles, D. J., van der Sande, M., Zaman, S., Touray, E., Hossin, S., Adetifa, J., Palmero, M., Burl, S., Jeffries, D., Rowland-Jones, S., Flanagan, K., Jaye, A., & Whittle, H. (2012). Immunological impact of an additional early measles vaccine in Gambian children: Responses to a boost at 3 years. *Vaccine*, *30*(15), 2543–2550. 10.1016/j.vaccine.2012.01.083 22314136 PMC3401374

[CIT0023] Ogutu, J. O., Francis, G. M., Kamau, D. M., Owiny, M. O., Oyugi, E. O., & Ettyang, G. K. (2023). Factors associated with low coverage of the second dose of measles containing vaccine among children aged 19–59 months, Alego-Usonga sub-county, Kenya, 2020. *Journal of Interventional Epidemiology and Public Health*, *6*(1). https://www.afenet-journal.net/content/article/6/1/full

[CIT0024] Okwaraji, Y. B., Mulholland, K., Schellenberg, J., Andarge, G., Admassu, M., & Edmond, K. M. (2012). The association between travel time to health facilities and childhood vaccine coverage in rural Ethiopia. A community-based cross-sectional study. *BMC Public Health*, *12*(1), 1–9. 10.1186/1471-2458-12-476 22726457 PMC3439329

[CIT0025] Onoja, A., & Ajagbe, O. (2019). Measles in developing countries, 1–10. Retrieved September 26, 2021, from http://www.who.int/immunization/monitoring_surveillance/burden/vpd/WHO_SurveillanceVaccinePreventable_11_Measles_R2.pdf

[CIT0026] Patel, M. K., Goodson, J. L., Alexander, J. P., Kretsinger, K., Sodha, S. V., Steulet, C., Gacic-Dobo, M., Rota, P. A., McFarland, J., Menning, L., Mulders, M. N., & Crowcroft, N. S. (2020). Progress toward regional measles elimination—worldwide, 2000–2019. *MMWR. Morbidity and Mortality Weekly Report*, *69*(45), 1700–1705. 10.15585/mmwr.mm6945a6 33180759 PMC7660667

[CIT0027] Perry, R. T., Gacic-Dobo, M., Dabbagh, A., Mulders, M. N., Strebel, P. M., Okwo-Bele, J.-M., Rota, P. A., & Goodson, J. L. (2014). Progress toward regional measles elimination—worldwide, 2000–2013. *Morbidity and Mortality Weekly Report*, *63*(45), 1034. 25393223 PMC5779499

[CIT0028] PTRoSRaM-A. Retrieved November 18, 2021, from http://www.prismastatement.orgPRISMA/Statement/Checklist.aspx

[CIT0029] Russo, G., Miglietta, A., Pezzotti, P., Biguioh, R. M., Bouting Mayaka, G., Sobze, M. S., Stefanelli, P., Vullo, V., & Rezza, G. (2015). Vaccine coverage and determinants of incomplete vaccination in children aged 12–23 months in Dschang, West Region, Cameroon: A cross-sectional survey during a polio outbreak. *BMC Public Health*, *15*(1), 1–11. 10.1186/s12889-015-2000-2 26156158 PMC4496879

[CIT0030] Strebel, P., Cochi, S., Grabowsky, M., Bilous, J., Hersh, B. S., Okwo-Bele, J. M., Hoekstra, E., Wright, P., & Katz, S. (2003). The unfinished measles immunization agenda. *The Journal of Infectious Diseases*, *187*(Supplement_1), S1–S7. 10.1086/368226 12721885

[CIT0031] Sugishita, Y., Kurita, J., Akagi, T., Sugawara, T., & Ohkusa, Y. (2019). Determinants of vaccination coverage for the second dose of measles-rubella vaccine in Tokyo, Japan. *The Tohoku Journal of Experimental Medicine*, *249*(4), 265–273. 10.1620/tjem.249.265 31852852

[CIT0032] Tadesse, A. W., Sahlu, D., & Benayew, M. (2022). Second-dose measles vaccination and associated factors among under-five children in urban areas of North Shoa Zone, Central Ethiopia, 2022. *Frontiers in Public Health*, *10*, 1029740. 10.3389/fpubh.2022.1029740 36568740 PMC9780268

[CIT0033] Tatsuo, H., Ono, N., Tanaka, K., & Yanagi, Y. (2000). SLAM (CDw150) is a cellular receptor for the measles virus. *Nature*, *406*(6798), 893–897. 10.1038/35022579 10972291

[CIT0034] Teshale, A. B., & Amare, T. (2023). Exploring spatial variations and the individual and contextual factors of uptake of measles-containing second dose vaccine among children aged 24 to 35 months in Ethiopia. *PLoS One*, *18*(1), e0280083. 10.1371/journal.pone.0280083 36598928 PMC9812309

[CIT0035] UNAIDS JUNPoHA. (2014). *90–90-90: An ambitious treatment target to help end the AIDS epidemic*. Accessed 2019, http://www.unaids.org/sites/default/files/media_asset/90-90-90_en_0.pdf

[CIT0036] WHO/Europe. (2015). *European vaccine action plan*, 98. Retrieved September 26, 2021, from http://www.euro.who.int/__data/assets/pdf_file/0007/255679/WHO_EVAP_ UK_v30_WEBx.pdf

[CIT0037] Wiysonge, C. S., Uthman, O. A., Ndumbe, P. M., & Hussey, G. D. (2012). Individual and contextual factors associated with low childhood immunization coverage in sub-Saharan Africa: A multilevel analysis. *PLoS One*, *7*(5), e37905. 10.1371/journal.pone.0037905 22662247 PMC3360654

[CIT0038] Wolfson, L. J., Grais, R. F., Luquero, F. J., Birmingham, M. E., & Strebel, P. M. (2009). Estimates of measles case fatality ratios: A comprehensive review of community-based studies. *International Journal of Epidemiology*, *38*(1), 192–205. 10.1093/ije/dyn224 19188207

[CIT0039] World Health Organization. (2002). WHO-UNICEF joint statement on strategies to reduce measles mortality worldwide. *Weekly Epidemiological Record= Relevé épidémiologique hebdomadaire*, *77*(27), 224–228. 12125242

[CIT0040] World Health Organization. (2009a). Measles vaccine: WHO position paper. *Weekly Epidemiological Record*, *84*, 349–360. https://apps.who.int/iris/bitstream/handle/10665/241403/WER8435_349-360.PDF19714924

[CIT0041] World Health Organization. (2009b). WHO position on measles vaccines. *Vaccine*, *27*(52), 7219–7221. 10.1016/j.vaccine.2009.09.116 19833246

[CIT0042] World Health Organization. (2010). WHO/UNICEF estimates of national immunization coverage. *World Health Organization*, 2–24. 10.2471/BLT.08.053819 PMC2704038 19649368

[CIT0043] World Health Organization. (2012). Global measles and rubella strategic plan: 2012.

[CIT0044] World Health Organization. (2017a). Global Health Observatory (GHO) data> measles-containing vaccine first-dose (MCV1) immunization coverage among 1-year-olds.

[CIT0045] World Health Organization. (2017b). Measles vaccines. *Weekly Epidemiological Record*, *92*(17), 313–324.

[CIT0046] World Health Organization. (2017c). Measles vaccines: WHO position paper, April 2017. *Weekly Epidemiological Record*, *92*, 205–228.28459148

[CIT0047] World Health Organization. (2019). Measles vaccines: WHO position paper, April 2017 – recommendations. *Vaccine*, *37*(2), 219–222. 10.1016/j.vaccine.2017.07.066 28760612

[CIT0048] World Health Organization. (2022). Eastern mediterranean, vaccine preventable diseases and immunization, progress towards measles elimination in the region.

[CIT0049] Zealiyas, K. (2020). STD & HIV/AIDS 2018: Viral load suppression status and associated factors among patient on antiretroviral treatment in Ethiopia – Kidist Zealiyas – Ethiopian Public Health Institute, Ethiopia. *6*(2).

